# Population-Wide Duchenne Muscular Dystrophy Carrier Detection by CK and Molecular Testing

**DOI:** 10.1155/2020/8396429

**Published:** 2020-09-27

**Authors:** Shuai Han, Hong Xu, Jinxian Zheng, Junhui Sun, Xue Feng, Yue Wang, Wen Ye, Qing Ke, Yanwei Ren, Shulie Yao, Songying Zhang, Jianfen Chen, Robert C. Griggs, Zhengyan Zhao, Ming Qi, Michele A. Gatheridge

**Affiliations:** ^1^Department of Cell Biology and Medical Genetics, School of Medicine, Zhejiang University, Hangzhou 310000, China; ^2^Department of Obstetrics, Zhejiang Provincial People's Hospital, People's Hospital of Hangzhou Medical College, No. 158, Shangtang Road, Hangzhou, Zhejiang Province, China; ^3^DIAN Diagnostics, Hangzhou 310000, China; ^4^Assisted Reproduction Unit, Department of Obstetrics and Gynecology, Department of Laboratory Medicine, Sir Run Run Shaw Hospital, Zhejiang University School of Medicine, Key Laboratory of Reproductive Dysfunction Management of Zhejiang Province, Hangzhou 310000, China; ^5^Hangzhou Family Planning Publicity and Technology Guidance Station/Hangzhou Health Service Center for Children and Women, Hangzhou 310000, China; ^6^Department of Neurology, The First Affiliated Hospital, Zhejiang University School of Medicine, Hangzhou 310000, China; ^7^Department of Obstetrics and Gynecology, Sir Run Run Shaw Hospital, Zhejiang University School of Medicine, Hangzhou 310000, China; ^8^Department of Neurology, University of Rochester School of Medicine and Dentistry, Rochester, New York 14642, USA; ^9^Department of Child Health Care, Children's Hospital Zhejiang University School of Medicine, Hangzhou 310000, China; ^10^Department of Pathology and Laboratory Medicine, University of Rochester Medical Center, Rochester, New York 14642, USA

## Abstract

Carrier screening of Duchenne muscular dystrophy (DMD) has not been widely evaluated. To identify definite DMD female carriers prior to or in early pregnancy, we studied a large population of reproductive age females and provided informed reproductive options to DMD carriers. 37268 females were recruited from the Hangzhou Family Planning Publicity and Technology Guidance Station/Hangzhou Health Service Center for Children and Women, Hangzhou, China, between October 10, 2017, and December 16, 2018. CK activity was measured with follow-up serum *DMD* genetic testing in subjects with hyperCKemia, defined as CK > 200 U/L. The calculated upper reference limit (97.5^th^ percentile) of serum creatine kinase (CK) for females aged 20-50 years in this study was near the reference limit recommended by the manufacturer (200 U/L), above which was defined as hyperCKemia. 427 females (1.2%) harbored initially elevated CK, among which 281 females (response rate of 65.8%) accepted CK retesting. *DMD* genetic testing was conducted on 62 subjects with sustained serum CK > 200 U/L and 16 females with a family history of DMD. Finally, 6 subjects were confirmed to be DMD definite carriers. The estimated DMD female carrier rate in this study was 1 : 4088 (adjusting for response rate), an underestimated rate, since only 50% to 70% of DMD female carriers manifest elevated serum CK, and carriers in this study may have been missed due to lack of follow-up or inability to detect all DMD pathogenic variants by current genetic testing.

## 1. Introduction

Duchenne muscular dystrophy (DMD) (MIM **#**310200) is a lethal degenerative neuromuscular disease, characterized by progressive muscular weakness, leading to motor delays, loss of ambulation, respiratory impairment, and cardiomyopathy, due to the loss of the protein dystrophin. Becker muscular dystrophy (BMD) (MIM #300376) is similar to DMD but has a more benign phenotype due to preservation of the dystrophin reading frame. The prevalence of DMD has been reported as 1 : 5000 male births [1]. DMD/BMD is inherited in an X-linked recessive manner, and the pathogenic *DMD* variant usually is passed on from a heterozygous mother (66%), or spontaneous mutations (33%). A typical laboratory finding in all DMD patients, elevated plasma creatine phosphokinase (CK) concentration due to progressive muscular damage, occurs in about 50% to 70% of asymptomatic heterozygous female DMD carriers [2, 3]. This statistic was confirmed in a presurvey conducted by our group, where we recruited 32 definite DMD carriers from the Zhejiang Muscular Disease (MD) care center and found that 50% of confirmed DMD carriers had increased CK activity (Table S1).

Recent advances in the treatment of boys with DMD hold promise to improving the prognosis of the disease [4–8]. Corticosteroids, prednisone, and deflazacort improve strength, pulmonary function, and timed motor function, as well as delay loss of ambulation in boys with DMD [9, 10]. Deflazacort, initially approved by the United States Food and Drug Administration (FDA) in 2017, for treatment of children with DMD ages 5 and older, was recently approved by the FDA for use in children ages 2-5 years old [11]. The first molecular-based treatment, Eteplirsen, an exon skipping therapy specifically aimed at patients with exon 51 variants, was approved by the FDA in 2016, under an accelerated pathway, to further study its benefit on disease progression [12]. Additionally, gene therapies which introduce truncated dystrophin converting DMD into the milder BMD phenotype are in active trials [13, 14]. In sum, all treatments require earlier intervention to achieve maximal benefit, justifying the exploration of DMD carrier screening programs, in combination with newborn screening programs [15]. In the past, DMD carrier screening studies have been completed on a relatively small scale [3, 16]. Accordingly, we aimed to study a large population of reproductive age females in Hangzhou, China, to identify asymptomatic female DMD carriers, regardless of their family history, and provide reproductive options to assess the effect DMD carrier screening will have on female preconception or pregnancy choices.

## 2. Materials and Methods

### 2.1. Subjects

The DMD carrier screening protocol in Hangzhou, China, was initiated on October 10, 2017. Participants were female who intended to bear children or were currently pregnant for no more than 3 months and were mobilized by the Hangzhou Family Planning Publicity and Technology Guidance Station/Hangzhou Health Service Center for Children and Women, Hangzhou, Zhejiang province, China. The commission covers most of the females in Hangzhou city. Potential participants freely attended an education seminar with group genetic counseling and received pamphlets regarding DMD carrier screening. All the participants were fully informed of the screening workup, including testing plasma CK concentration and potential DMD gene testing, assayed through their peripheral blood. Informed consent was obtained for all participants enrolled, and all participants were notified of the results of their screening. Asymptomatic female DMD carries are defined as genetically definite manifesting carriers without skeletal muscle weakness (e.g., muscle weakness, walking with frequent falling, abnormal gait, difficulty running, jumping, or climbing stairs) or cardiac symptoms. All subjects were asked to fill out a questionnaire to confirm the absence of any skeletal muscle symptoms or cardiac abnormalities. Confirmed carriers of *DMD* pathogenic variants were offered professional genetic counseling by a certified genetic counselor. Depending on their pregnancy status, participants were also provided several reproductive choices: preimplantation genetic diagnosis or prenatal genetic testing through chorionic villus, amniotic fluid sampling, or umbilical cord blood, during different gestational weeks.

### 2.2. Screening Approach

**Figure 1:**
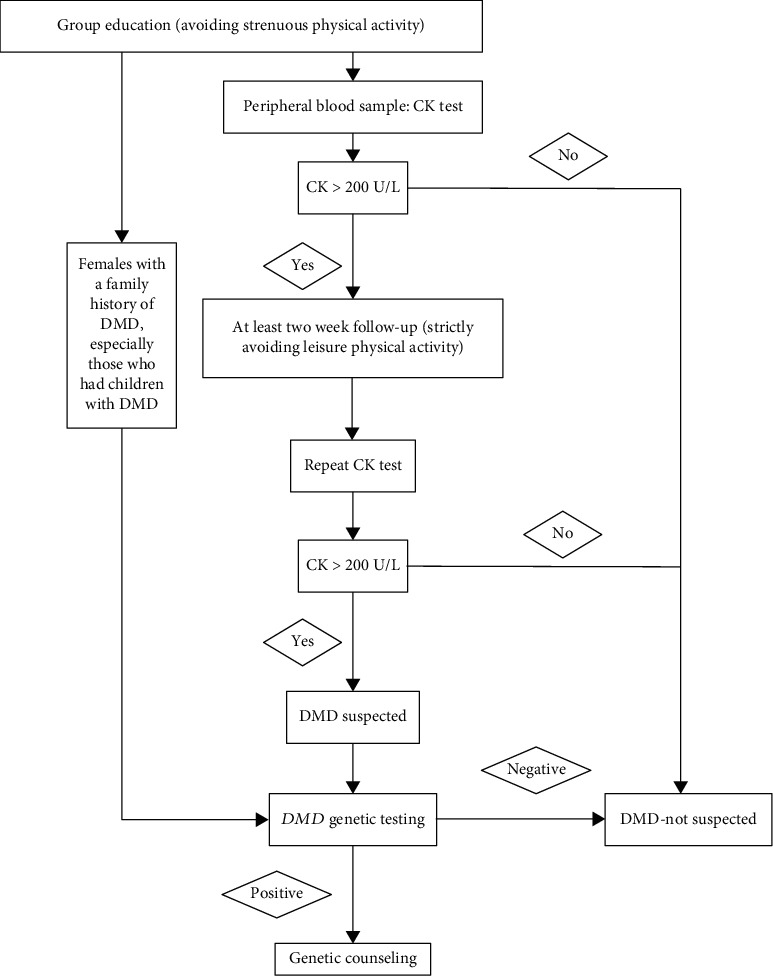
Screening workup of asymptomatic creatine kinase elevation in females.

The screening process was approved by the local ethics committee, and subjects were fully informed. There was no cost for any participant.  Figure 1 shows the full protocol. For subjects identified to have a normal plasma CK concentration (40-200 U/L), no action was taken. If the CK value was above the cut-off of 200 U/L, a repeat CK assay was requested after 14 days during which time the subject was asked to avoid strenuous exercise. If the repeat CK remained above 200 U/L, the *DMD* gene was tested for gross deletion/duplication and point mutation/small deletion/small duplication by Multiplex Ligation-dependent Probe Amplification (MLPA). Next-generation sequencing (NGS) of the whole *DMD* gene for point mutations and small deletion/insertions would be performed to all the MLPA-negative samples, as described below (Table S2). Moreover, for women with a family history of DMD, especially those who had children with DMD, genetic testing was implemented regardless of their CK level.

### 2.3. Plasma CK Measurement

The peripheral blood collected was transported immediately to the laboratory, and concentration of CK activity was detected by Roche Cobasc 701/702 using an enzymatic rate method. The laboratory reference range for this assay is 40-200 U/L for women, the central 95% of observations in caucasians [17]. Usually, the concentration of plasma creatine kinase is below 200 U/L in normal females, except for the following conditions: neuromuscular disease, strenuous physical activity, cardiovascular diseases, tumors, trauma, statins, endocrine disorders, and infectious hepatitis.

### 2.4. DNA Preparation

In participants who had a family history of DMD and were found to have an elevated serum CK (>200 U/L) on initial and repeat testing, genomic DNA was isolated from white blood cells, by QIAamp DNA Blood Mini kit (Qiagen), and used in MLPA and NGS per the protocol below. In one subject, case 2 (described in more detail in the discussion), 20 ml of amniotic fluid was taken during amniocentesis and the DNA of the fetal amniotic fluid cells was extracted using the QIAamp DNA Mini kit following the manufacturer's recommendation, which was used in PCR-based Sanger sequencing.

### 2.5. Multiplex Ligation-Dependent Probe Amplification (MLPA)

Large deletions/duplications of one or more exons in the *DMD* gene account for about 65% to 80% of *DMD* variants [18, 19]. MLPA is performed to detect gross deletion and duplication of the *DMD* gene, using SALSA MLPA P034 and P035 probemixe kit (available commercially MRC Holland, Netherlands). Coffalyser.Net software was used for data analysis in combination with the appropriate lot-specific MLPA Coffalyser sheet. The whole process was performed strictly according to the manufacturer's instructions [20]. Positive and normal DNA samples were used for controls in every testing.

### 2.6. Next-Generation Sequencing Based on Multiplex PCR

Point mutations and small deletion/insertions can be found in the remaining 20% to 35% of patients, which can be detected using a high-throughput detection technique [18, 19]. All 79 exons and the exon-intron boundaries of the *DMD* gene were studied through Multiple PCR amplification (GenSeizer DMD Panel Mix, morgene, China) and direct sequencing (Illumina MiSeq).

### 2.7. Validation by PCR-Based Sanger Sequencing

Primers (F-AGCCAACACACACATCCTTT/R-ACTTGGGACTCTGCAATTTGT) for DMD c.10364dupT and primers (F-TTGCTTTGTGTGTCCCATGC/R- ACTCATTACCGCTGCCCAAA) for *DMD* c.7555G>A were designed according to the *DMD g*ene sequence, to further validate the next-generation sequencing results. The extracted genomic DNA was amplified per the following procedure: 95°C 5 minutes, 30× (95°C 30 seconds, 60°C 30 seconds, and 72°C 30 seconds), 72°C 10 minutes. PCR products were sequenced and then analyzed using the software CHROMAS.

### 2.8. Bioinformatics Analysis

SIFT, Polyphen-2, Mutation Assessor, and PROVEAN (Protein Variation Effect Analyzer) were used to predict whether an amino acid substitution could influence the protein function.

## 3. Results

**Figure 2:**
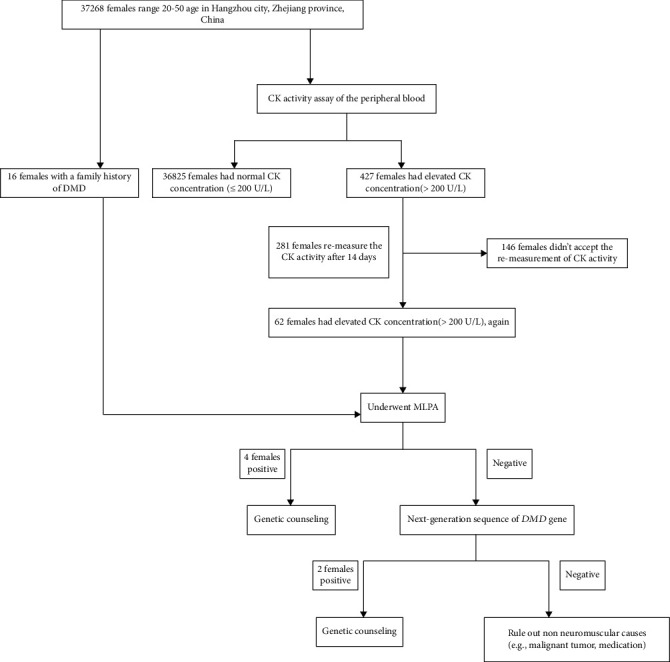
Results of screening program for DMD carriers in Hangzhou city, Zhejiang province, China, during October 10, 2017, to December 16, 2018.

**Figure 3:**
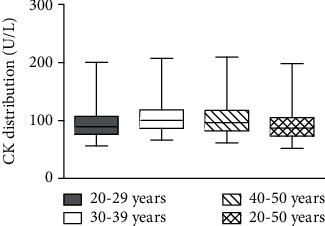
CK characteristics of the study population by age. Bars indicate 2.5^th^ and 97.5^th^ percentile.

The results of the Hangzhou DMD Carrier Screening Program are shown in Figure 2. Between October 10, 2017, and December 16, 2018, the plasma CK of 37268 females was screened. The CK characteristics of the study population are depicted in Figure 3. After deleting the errors and outliers, the estimated 97.5^th^ percentile CK for subjects, 20-29 years, 30-39 years, and 40-50 years, is 201 U/L, 208 U/L, and 215 U/L, respectively (Figure 3). We summarize that the actual central 95% distribution of serum CK values in Chinese women aged 20-50 years is 36-207 U/L (Figure 3), near to the reference interval (40-200 U/L) recommended by the manufacturer. Thus, CK values > 200 U/L were defined as hyperCKemia in this cross-sectional study and 427 females (1.2%) were identified to have an initially elevated plasma CK (>200 U/L). After notification, 281 females (response rate of 65.8%) accepted CK retesting; among whom, 62 females (1 : 396 adjusting for response rate) had sustained elevated serum CK, and then, genetic testing was carried out. The other 219 were identified to have a transient raised CK (Table S3). For 16 females with a family history of DMD, genetic testing was implemented regardless of their CK level. MLPA and NGS of the *DMD* gene identified 6 definite DMD carriers with clear pathogenic variants (3 of the 16 subjects with positive family history and 3 of the 62 subjects with negative family history) (Table S2). Four cases had deletions and/or duplications of *DMD* gene, mostly located in a hotspot mutation region (exons 44-55), one case carried a previous reported pathogenic missense variant, and a novel deleterious frameshift pathogenic variant was found in one case. Overall, the estimated DMD female carrier rate in this study is 1 : 4088, adjusting for response rate. In follow-up, all the carriers identified by this screening denied muscular weakness or cardiac symptoms.

Below, we describe these cases and the outcome carrier screening had on conception or pregnancy, if applicable.

### 3.1. Positive DMD Family History: Cases 1, 2, and 3

**Figure 4:**
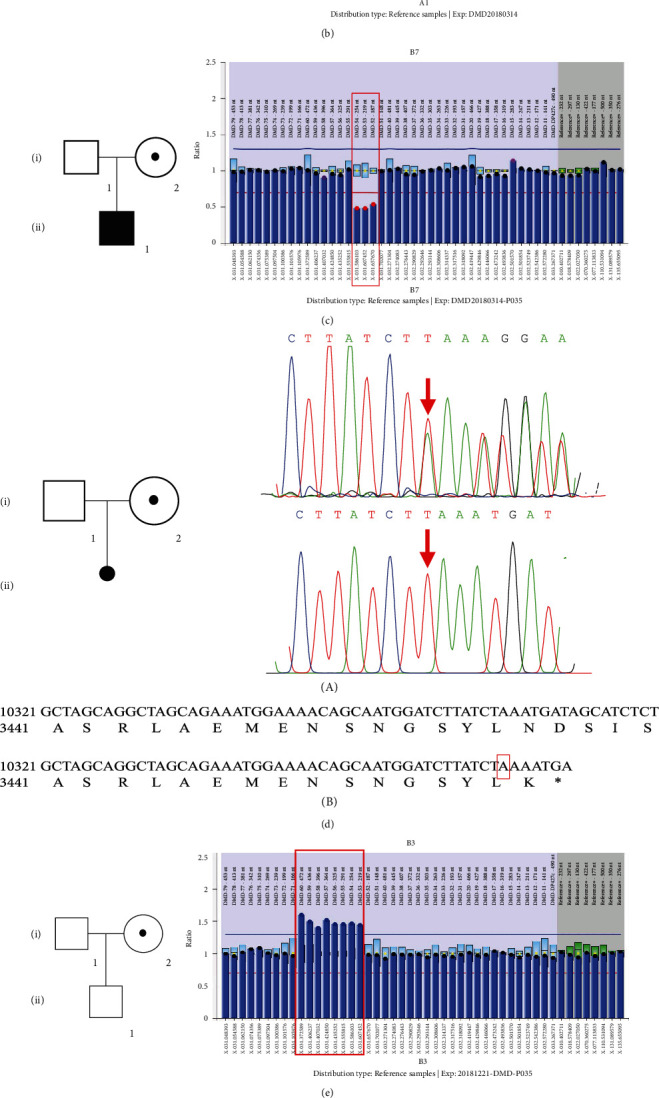
Pedigrees (left) and the results of *DMD* gene testing (right). (a) Pedigree of the family of carrier 1 (III2). The carrier status is indicated by a circle with a dot, sloid symbols (affected), clear symbols (unaffected). The result of MLPA analysis on the right shows exon 44del in *DMD* gene (the horizontal axis represents exon number of *DMD*, and the vertical axis shows the relative peak area ratios compared to the mean of control samples). (b) Pedigree of the family of carrier 2 (I2). The carrier status is indicated by a circle with a dot, sloid symbols (affected), clear symbols (unaffected). The result of MLPA on the right shows exon 43del in DMD gene. (c) Pedigree of the family of carrier 3 (I2). The carrier status is indicated by a circle with a dot, sloid symbols (affected), clear symbols (unaffected). The result of MLPA on the right shows exon 52-54del in *DMD* gene. (d) A: pedigree of the family of carrier 4 (I2). The carrier status is indicated by a circle with a dot, abortion (a dot), clear symbols (unaffected). PCR-based Sanger sequencing on the right validates the results of next generation sequencing: the arrow represents the carrier (I2) with 1 heterozygous insertion variant c.10364 dup T of exon 73 (upper right) as well as the same but hemizygous variant in the aborted embryo (lower right). B: the amino acid sequences of the wild type and mutant were subjected to DNAMAN, revealing that the variant produced a truncated dystrophin. (e) Pedigree of the family of carrier 5 (I2). The carrier status is indicated by a circle with a dot, clear symbols (unaffected). The result of MLPA on the right shows exon 52_60dup in *DMD* gene. (f) A: pedigree of the family of carrier 6 (I2). The carrier status is indicated by circle with a dot, clear symbols (unaffected). PCR-based Sanger sequencing on the right validates the results of next generation sequencing: the arrow represents the carrier (I2) with 1 heterozygous missense variant c.7555G>A of exon 52. (b) Multiple species alignment analysis showed the high evolution conservation of amino acid sequence at the missense site.

Case 1 is a female, 41 years old, previously delivered a 12-year-old healthy son, planning to have a second child. Upon further personal and family history, obtained after genetic testing, the subject noted a positive family history for DMD, an affected uncle, who had died years prior (Figure 4(a)). MLPA detected a deletion of exon 44, which has been recorded to be deleterious in the HGMD database (http://www.hgmd.cf.ac.uk), manifesting that she is a definite asymptomatic carrier (Figure 4(a)) [21]. We offered genetic counseling to her and her family, explaining the progression of the disease and future reproductive choices.

Cases 2 and 3 are females, 42 years old and 31 years old, both previously delivered affected DMD boys (12 years old and 7 years old, respectively, at the time of the study). Per the screening program protocol, MLPA detected a deletion of exon 43 (case 2) and exons 52-54 (case 3), which have been described in multiple unrelated individuals with DMD, and are expected to cause loss of normal protein function through either truncation or nonsense-mediated mRNA decay [21, 22]. With the exception of their sons, the subjects had no other family history of DMD or relevant personal history of neuromuscular or cardiac symptoms. Both subjects were offered genetic counseling and informed of preimplantation genetic diagnosis or prenatal diagnosis for potential future pregnancies.

### 3.2. Negative DMD Family History: Cases 4, 5, and 6

Case 4 is a female, 23 years old, 12 weeks pregnant. Initial CK was 350 U/L with repeat CK 420 U/L. Next-generation sequencing revealed a c.10364dup (p. Asn3456fs) variant in the *DMD* gene, confirmed by Sanger sequencing (Figure 4(d)). This variant could lead to protein truncation and has not been reported before (Figure 4(d)). Through a detailed family history enquiry, we learned that the subject was adopted by her foster parents, so, we cannot identify whether the variant is de novo or not (Figure 4(d)). According to the American College of Medical Genetics guidelines, the frameshift variant can be classified as a likely pathogenic variant. After an hour of genetic counseling session, the subject chose prenatal genetic testing. The amniotic fluid of the pregnant woman was extracted, and Sanger sequencing showed that the boy fetus inherited the pathogenic variant. After two hours with a professional genetic counselor at the Zhejiang MD Care Center, the subject chose to abandon the fetus, given the young gestational age and potential severe outcome to the baby boy and the whole family. Obstetricians and Gynecologists implemented induced abortion the following week.

Cases 5 and 6 are females, 48 years old and 30 years old, were extremely surprised when they were informed of the positive result of genetic testing, based on that they had already delivered healthy boys (12 years old and 8 years old, respectively, at the time of the study). The duplication of exon 53-60 in *DMD* gene (carried by Case 5) has been identified in many unrelated patients (http://www.umd.be/DMD/4DACTION/WSEARCH) [23]. Case 6 carried a missense variant c.7555G>A, p. D2519N in *DMD* gene previously reported as a pathogenic variant [24].

Of the other 56 females with sustained increased serum creatine kinase but negative results of *DMD* genetic testing, medications and diseases such as malignancies were the most common cause of hyperCKemia.

## 4. Discussion

This is the first systematic and comprehensive CK screening program for DMD carriers, demonstrating the feasibility of screening asymptomatic DMD carriers through serum CK measurement combined with *DMD* genetic testing. Elevated serum CK exists in approximately 50% to 70% of asymptomatic heterozygous female DMD carriers [3, 18]. Using the serum CK screening protocol described here, we successfully identified 6 definite DMD carriers. Previous CK studies in the detection of female carriers of DMD were reported in 1971, and again in 1998, but neither study focused on carrier screening on a large scale [3, 16]. Heterozygous females, who are usually asymptomatic, have a 50% chance of transmitting the *DMD* pathogenic variant in each pregnancy, and a 25% chance of giving birth to an affected boy [18]. Current carrier screening only occurs in the setting of a family history of DMD, missing many spontaneous cases resulting from unsuspected and new maternal variants [25]. Carrier screening before or early in pregnancy will allow carrier females to receive genetic counseling and be informed of fertility choices and recurrent risk. Moreover, carrier screening will help carriers prepare for the possibility of manifesting DMD-related symptoms later on in life. At least 2.5% to 10% of DMD female carriers manifest muscle weakness [26]. Asymptomatic carriers also have an increased risk of developing cardiomyopathy with age. The clinical guidelines in Europe and the United States recommended that adult dystrophinopathy carriers should undergo echocardiography every 5 years [27]. Furthermore, female relatives of positive carriers should be recommended for genetic testing in order to evaluate their carrier status. An important point from this study is that varied CK values were identified even within the same DMD variant (Table S1). As known, X-chromosome inactivation (XCI) is a random process which occurs early in embryogenesis resulting in females being functionally hemizygous for the majority of the genes on the X chromosome. Matthews et al. found that the proportion of XCI can vary not only between different tissues but even between different skeletal muscles of the same subject [28]. So, for nonmanifesting or asymptomatic carriers of DMD, the proportion of the preferential inactivation (usually nearing 50% to 80%) could also vary even among different subjects harboring the same variant of DMD [29], even excluding any clinical, electrophysiological, or pathological evidence of neuromuscular diseases.

Among the 6 definite variants identified during the carrier screening program, the D2519N missense variant has been reported previously as pathogenic in a DMD male [24] and benign without further interpretation in the ClinVar database. It was not observed in approximately 6,500 individuals of European and African American ancestry in the National Heart, Lung, and Blood Institute (NHLBI) Exome Sequencing Project, and the substitution occurs at a position that is conserved across species (Sorting Intolerant From Tolerant and PolyPhen-2 predicted it deleterious and probably damaging). Furthermore, we assumed that it might be de novo in the family given that there was no history of neuromuscular diseases. Accordingly, we classified D2519N as likely pathogenic.

It is useful to compare this carrier screening protocol with that adopted in the newborn screening (NBS) programs for DMD since 1975 [1, 30–39]. Gatheridge et al. reviewed these screening programs, in which, bloodspot/serum CK screened at birth or at early age, before the onset of DMD symptoms, was studied to avoid the anxiety associated with the DMD diagnostic delay, to enable families to plan for a future with a child with disability, and to give families reproductive choices in future pregnancies [15]. All of the early DMD screening programs used a 3-step testing approach, similar to the one our program adopted, designed to reduce the number of false positive results that might occur with single CK testing [15]. CK levels transiently rise within 24 hours of strenuous physical activity such as exercise or heavy manual labor, then slowly decline over the next 7 days. In assessing asymptomatic CK elevation, the test should be repeated after 7 days without exercise [17]. In our study, after 2 CK measurements, at least 7 days apart, 62 females had sustained elevated serum CK, a reasonable number for genetic analysis. More recent newborn screening programs in Ohio, USA, and Hangzhou, China, have adopted a 2-tier approach, testing serum CK and *DMD* gene testing on the same dried blood spot [1, 40]; although likely more costly, this method could be considered in future carrier screening programs. The direct cost of CK screening is about one US dollar per test. *DMD* genetic testing is about $400 US dollars per test (MLPA and NGS), with a total lab direct cost for this program of about $63,000 US dollars. The cost of education material printing, seminar, physician genetic counseling time, project management, etc. was not included.

The NBS program in China is screening both genders, which is recommended, and thus, some carriers may be identified, however, since DMD newborn screening programs typically have much higher CK cut-offs, to limit false positives while increasing false negatives; many carriers in these programs may be missed, proving the importance of simultaneous DMD carrier screening programs.

For severe and some untreatable diseases including cystic fibrosis, sickle cell disease, DMD, and tuberous sclerosis, NBS and carrier screening programs have evolved through NGS such as whole-genome, exome, or gene-panel sequencing, reducing the total number of tests and time cost. By evaluating for single-nucleotide variants and small insertions/deletions in 163 NBS genes, Solomon et al. summarized that incorporating whole genome sequencing (WGS) into NBS yielded fewer false positives and detected some conditions not amenable to conventional NBS, providing precise molecular diagnoses for Mendelian disorders [41]. Kruszka et al. demonstrated that WES-based techniques could successfully be used as a screening test for large genomic copy number variations (CNVs) of Turner syndrome, in NBS [42]. Parent Project Muscular Dystrophy (PPMD), which initiated a national Duchenne Newborn Screening (DNBS) effort in December 2014 in the United States, is trending towards utilization of NGS panels to detect DMD and other muscular dystrophy-related gene variants [43]. NGS also improves thalassemia carrier screening for both sensitivity and specificity among premarital adults in the Dai nationality, China [44].

In addition to identifying DMD female carriers, this study also shed light on the CK variation in Chinese women aged 20-50 years. We were not able to analyze the CK distribution of pregnant participants, due to the small sample size (only 3 subjects). Montfrans et al. suggested that the general population, including people of European, South Asian, and African ancestry, often show relatively high CK activities, with CK levels in the Chinese population slightly higher than the mean [45]. HyperCKemia, defined by the European Federation of Neurological Societies (EFNS), is a CK 1.5 times the upper limit of normal. So, the reference range, 40-200 U/L (the central 95% of observations in Caucasians), for normal CK provided by the CK assay manufacturer can be adopted in this cross sectional study [17]. There are many causes of hyperCKemia besides DMD, such as non-DMD muscle disorders, medications (statins, fibrates, antiretrovirals, beta-blockers, clozapine, angiotensin II receptor blockers, hydroxychloroquine, isotretinoin, and colchicine), drugs, strenuous muscle exercise, trauma, surgery, toxins, endocrine disease, viral illness, metabolic conditions, chronic cardiac disease, tumor, malignant hyperthermia syndrome, obstructive sleep apnea, neuroacanthocytosis syndromes, and macro-CK [46]. Retesting serum CK is a good way to discriminate those transient elevation cases due to heavy manual labor or strenuous exercise, which can largely not only reduce false positive results but also save subsequent *DMD* gene testing cost. Moreover, Lilleng et al. have identified that persistent hyperCKemia (CK values ≥ 210 U/L in women ≥ 400 U/L in men < 50 years and ≥280 U/L in men ≥ 50 years) actually exists in 1.3% of the normal population [47]. In our study, 1.1% showed persistent hyperCKemia, the proportion may be higher if the reexamination rate can be improved. There may also be some risk factors we did not identify because of the limitation of disease screening scope and relatively short follow-up. False negative results are inevitable considering that the elevated CK level cannot be detected in all definite DMD carriers.

Previous studies have shown that most people with idiopathic elevated CK remain asymptomatic, as seen in this study [48]. In addition, hyperCKemia-related diseases screened may not be limited to DMD and could include other muscular diseases, especially those coexisting with DMD [3, 49–53]. This cohort provides a good base for further investigating those subjects with mildly elevated CK but whom *DMD* gene variant testing is negative.

Our approach to the screening of DMD female carriers has some limitations. First, as known, only 50% to 70% of DMD female carriers show elevated serum CK activity; thus, a significant portion of DMD female carriers will be missed. Second, 65.8% of participants with an initial elevated CK, returned for CK rescreening. This may be due to lack of motivation, either they decided to not pursue more children or the outcome of testing would not change their fertility path. A survey of the actual reasons and adopted measures to raise the rate of follow-up is warranted. Third, MLPA and NGS could not detect all *DMD* pathogenic variants such as rare gene rearrangement and point mutations in deep intron regions. The difficulty of follow-up and the nonfull detection rate of the genetic testing emphasizes the importance of NBS, which has the potential to screen nearly every newborn with initial blood spot testing [1]. In patients suspected to have DMD/BMD but genetic testing is negative, a classical skeletal muscle biopsy for western blot and immunohistochemistry studies of dystrophin or genetic testing of more comprehensive neuromuscular diseases such as limb-girdle muscular dystrophy (LGMD), myofibrillar myopathy, desmin-related myofibrillar myopathy, and myotonic dystrophy can be considered [43].

## 5. Conclusion

In conclusion, identifying DMD carriers provides females more reproductive choices and will allow for prenatal identification of newborns affected by DMD to implement promising new treatments at birth or potentially prenatally. Limitations in carrier screening, using elevated CK exist, considering that about 30% to 50% of carriers do not have an elevated CK and 34.2% of the participant in this study did not return for follow-up CK testing. Despite these limitations, CK screening is and will continue to be essential for DMD NBS and carrier screening programs. This study proves that a large-scale DMD carrier screening program is possible; however, to be successful, follow-up care with professional genetic counseling and patient support groups is key components.

## Data Availability

The data used to support the findings of this study are included within the article.

## Supplementary Materials

Supplementary Materials Table S1: serum creatine kinase concentration of definite DMD carriers in pre-survey. Table S2: family history and DMD genetic testing actually done of 62 females with twice serum creatine kinase elevation. Table S3: serum creatine kinase concentration of 219 females with transient elevation
