# Supplementary material for: Population-Wide Duchenne Muscular Dystrophy Carrier Detection by CK and Molecular Testing
Source: Biomed Res Int. 2020 Sep 27;2020:8396429. doi: 10.1155/2020/8396429 (PMC7537677; doi:10.1155/2020/8396429)
Supplement: Supplementary Materials — Table S1: serum creatine kinase concentration of definite DMD carriers in pre-survey. Table S2: family history and DMD genetic testing actually done of 62 females with twice serum creatine kinase elevation. Table S3: serum creatine kinase concentration of 219 females with transient elevation [file 8396429.f1.docx]

**Table S1. Serum creatine kinase concentration of definite DMD carriers in pre-survey.**

**Table S2. Family history and *DMD* genetic testing actually done of 62 females with twice Serum creatine kinase elevation and 16 females with a family history of DMD**

**Table S3. Serum creatine kinase concentration of 219 females with transient elevation**

**Table S1.Serum creatine kinase concentration of definite DMD carriers**

**in pre-survey.**

| **Proven DMD carriers** | **Age (y)** | **NM transript number** | **Pathogenic mutation** | **Concentration of serum creatine kinase(U/L)** |
| --- | --- | --- | --- | --- |
| Patient 1 | 24 | NM_000109.4 | *DMD*c.5697dup | 206 |
| Patient 2 | 46 | NM_000109.4 | *DMD* ex1del | 353 |
| Patient 3 | 49 | NM_000109.4 | *DMD* ex1del | 371 |
| Patient 4 | 22 | NM_000109.4 | *DMD* ex1del | 174 |
| Patient 5 | 27 | NM_000109.4 | *DMD* ex45_53del | 239 |
| Patient 6 | 48 | NM_000109.4 | *DMD* ex3_7dup | 818 |
| Patient 7 | 42 | NM_000109.4 | *DMD* ex3_7dup | 180 |
| Patient 8 | 21 | NM_000109.4 | *DMD*c.5697dup | 544 |
| Patient 9 | 25 | NM_000109.4 | *DMD*c.5697dup | 108 |
| Patient 10 | 33 | NM_000109.4 | *DMD* ex3_25del | 904 |
| Patient 11 | 31 | NM_000109.4 | *DMD* ex3_25del | 61 |
| Patient 12 | 35 | NM_000109.4 | *DMD* ex8_13del | 1155 |
| Patient 13 | 45 | NM_000109.4 | *DMD* ex3_7del | 62 |
| Patient 14 | 33 | NM_000109.4 | *DMD* ex48_54del | 564 |
| Patient 15 | 27 | NM_000109.4 | *DMD* ex52_54del | 117 |
| Patient 16 | 33 | NM_000109.4 | *DMD* ex49_51del | 65 |
| Patient 17 | 33 | NM_000109.4 | *DMD* ex46_52del | 120 |
| Patient 18 | 26 | NM_000109.4 | *DMD* ex45_48del | 72 |
| Patient 19 | 28 | NM_000109.4 | *DMD* ex45_48del | 104 |
| Patient 20 | 37 | NM_000109.4 | *DMD* ex45_52dup | 289 |
| Patient 21 | 27 | NM_000109.4 | *DMD* ex49_51del | 183 |
| Patient 22 | 30 | NM_000109.4 | *DMD* c.8498delC | 252 |
| Patient 23 | 29 | NM_000109.4 | *DMD* c.8498delC | 773 |
| Patient 24 | 13 | NM_000109.4 | *DMD* c.4150G>T | 203 |
| Patient 25 | 27 | NM_000109.4 | *DMD* ex45del | 205 |
| Patient 26 | 29 | NM_000109.4 | *DMD* ex52_54del | 74 |
| Patient 27 | 28 | NM_000109.4 | *DMD* c.2248dup | 128 |
| Patient 28 | 35 | NM_000109.4 | *DMD* c.94-1G>A | 26 |
| Patient 29 | 32 | NM_000109.4 | *DMD* c.94-1G>A | 87 |
| Patient 30 | 42 | NM_000109.4 | *DMD* c.94-1G>A | 862 |
| Patient 31 | 31 | NM_000109.4 | *DMD* ex13_44del | 1853 |
| Patient 32 | 30 | NM_000109.4 | *DMD* ex45del | 56 |

**Table S2. Family history and *DMD* genetic testing actually done of 62 females with twice Serum creatine kinase elevation and 16 female with a family history of DMD.**

| **Number** | **Age (y)** | **Initial Serum CK(U/I)** | **Second Serum CK(U/I)** | **Family History** | ***DMD* Genetic Testing**  **(Done)** | ***DMD* Pathogenic**  **Variation** |
| --- | --- | --- | --- | --- | --- | --- |
|  | 39 | 256 | 280 | Negative | MLPA+NGS | / |
|  | 24 | 285 | 266 | Negative | MLPA+NGS | / |
|  | 25 | 365 | 3281 | Negative | MLPA+NGS | / |
|  | 27 | 313 | 251 | Negative | MLPA+NGS | / |
|  | 25 | 234 | 253 | Negative | MLPA+NGS | / |
|  | 23 | 400 | 388 | Negative | MLPA+NGS | / |
|  | 28 | 227 | 218 | Negative | MLPA+NGS | / |
|  | 41 | 228 | 391 | Negative | MLPA+NGS | / |
|  | 21 | 242 | 225 | Negative | MLPA+NGS | / |
|  | 27 | 371 | 345 | Negative | MLPA+NGS | / |
|  | 41 | 345 | 334 | Negative | MLPA+NGS | / |
|  | 29 | 255 | 316 | Negative | MLPA+NGS | / |
|  | 26 | 505 | 436 | Negative | MLPA+NGS | / |
|  | 39 | 340 | 430 | Negative | MLPA+NGS | / |
|  | 25 | 209 | 239 | Negative | MLPA+NGS | / |
|  | 23 | 1087 | 297 | Negative | MLPA+NGS | / |
|  | 25 | 213 | 1411 | Negative | MLPA+NGS | / |
|  | 36 | 245 | 257 | Negative | MLPA+NGS | / |
|  | 23 | 297 | 467 | Negative | MLPA+NGS | / |
|  | 49 | 337 | 318 | Negative | MLPA+NGS | / |
|  | 35 | 413 | 436 | Negative | MLPA+NGS | / |
|  | 25 | 352 | 331 | Negative | MLPA+NGS | / |
|  | 25 | 269 | 317 | Negative | MLPA+NGS | / |
|  | 32 | 241 | 308 | Negative | MLPA+NGS | / |
|  | 29 | 489 | 470 | Negative | MLPA+NGS | / |
|  | 26 | 205 | 258 | Negative | MLPA+NGS | / |
|  | 26 | 249 | 249 | Negative | MLPA+NGS | / |
|  | 22 | 308 | 253 | Negative | MLPA+NGS | / |
|  | 32 | 264 | 249 | Negative | MLPA+NGS | / |
|  | 26 | 395 | 394 | Negative | MLPA+NGS | / |
|  | 27 | 770 | 743 | Negative | MLPA+NGS | / |
|  | 25 | 559 | 469 | Negative | MLPA+NGS | / |
|  | 28 | 346 | 314 | Negative | MLPA+NGS | / |
| **Number** | **Age (y)** | **Initial Serum CK(U/I)** | **Second Serum CK(U/I)** | **Family History** | ***DMD* Genetic Testing**  **(Done)** | ***DMD* Pathogenic**  **Variation** |
|  | 24 | 1368 | 252 | Negative | MLPA+NGS | / |
|  | 27 | 234 | 523 | Negative | MLPA+NGS | / |
|  | 25 | 321 | 347 | Negative | MLPA+NGS | / |
|  | 23 | 316 | 307 | Negative | MLPA+NGS | / |
|  | 28 | 295 | 210 | Negative | MLPA+NGS | / |
|  | 44 | 280 | 488 | Negative | MLPA+NGS | / |
|  | 30 | 375 | 307 | Negative | MLPA+NGS | / |
|  | 25 | 284 | 221 | Negative | MLPA+NGS | / |
|  | 29 | 413 | 317 | Negative | MLPA+NGS | / |
|  | 34 | 299 | 320 | Negative | MLPA+NGS | / |
|  | 32 | 1018 | 921 | Negative | MLPA+NGS | / |
|  | 26 | 458 | 431 | Negative | MLPA+NGS | / |
|  | 29 | 275 | 232 | Negative | MLPA+NGS | / |
|  | 40 | 430 | 439 | Negative | MLPA+NGS | / |
|  | 34 | 215 | 703 | Negative | MLPA+NGS | / |
|  | 21 | 326 | 365 | Negative | MLPA+NGS | / |
|  | 38 | 242 | 231 | Negative | MLPA+NGS | / |
|  | 47 | 266 | 202 | Negative | MLPA+NGS | / |
|  | 26 | 412 | 434 | Negative | MLPA+NGS | / |
|  | 25 | 252 | 321 | Negative | MLPA+NGS | / |
|  | 27 | 499 | 672 | Negative | MLPA+NGS | / |
|  | 35 | 221 | 271 | Negative | MLPA+NGS | / |
|  | 36 | 380 | 493 | Negative | MLPA+NGS | / |
|  | 45 | 391 | 220 | Negative | MLPA+NGS | / |
|  | 41 | 330 | 449 | Negative | MLPA+NGS | / |
|  | 37 | 327 | 335 | Negative | MLPA+NGS |  |
|  | 23 | 350 | 420 | Negative | MLPA+NGS | *DMD* c.10364dup |
|  | 48 | 831 | 1567 | Negative | MLPA | *DMD* ex53_60dup |
|  | 30 | 203 | 230 | Negative | MLPA+NGS | *DMD* c.7555G>A |
|  | 22 | / | / | Positive(Son Affected) | MLPA+NGS | / |
|  | 34 | / | / | Positive(Uncle Affected) | MLPA+NGS | / |
|  | 21 | / | / | Positive(Uncle Affected) | MLPA+NGS | / |
|  | 30 | / | / | Positive(Uncle Affected) | MLPA+NGS | / |
|  | 27 | / | / | Positive(Uncle Affected) | MLPA+NGS | / |
|  | 29 | / | / | Positive(Uncle Affected) | MLPA+NGS | / |
| **Number** | **Age (y)** | **Initial Serum CK(U/I)** | **Second Serum CK(U/I)** | **Family History** | ***DMD* Genetic Testing**  **(Done)** |  |
|  | 28 | / | / | Positive(Uncle Affected) | MLPA+NGS | / |
|  | 41 | / | / | Positive(Uncle Affected) | MLPA+NGS | / |
|  | 33 | / | / | Positive(Uncle Affected) | MLPA+NGS | / |
|  | 31 | / | / | Positive(Uncle Affected) | MLPA+NGS | / |
|  | 34 | / | / | Positive(Uncle Affected) | MLPA+NGS | / |
|  | 25 | / | / | Positive(Uncle Affected) | MLPA+NGS | / |
|  | 32 | / | / | Positive(Uncle Affected) | MLPA+NGS | / |
|  | 41 | / | / | Positive(Uncle Affected) | MLPA | *DMD* ex44del |
|  | 42 | / | / | Positive(Son Affected) | MLPA | *DMD* ex43del |
|  | 31 | / | / | Positive(Son Affected) | MLPA | *DMD* ex52_54del |

**Table S3.Serum creatine kinase concentration of 219 females with transient elevation**

| **Number** | **Age (y)** | **Initial Serum CK(U/I)** | **Second Serum CK(U/I)** |
| --- | --- | --- | --- |
| 1 | 25 | 420 | 61 |
| 2 | 24 | 756 | 67 |
| 3 | 29 | 248 | 59 |
| 4 | 25 | 285 | 80 |
| 5 | 27 | 377 | 68 |
| 6 | 25 | 213 | 63 |
| 7 | 44 | 325 | 189 |
| 8 | 38 | 245 | 101 |
| 9 | 26 | 203 | 73 |
| 10 | 33 | 443 | 119 |
| 11 | 35 | 337 | 107 |
| 12 | 28 | 314 | 183 |
| 13 | 29 | 203 | 78 |
| 14 | 35 | 312 | 115 |
| 15 | 24 | 1001 | 70 |
| 16 | 42 | 250 | 190 |
| 17 | 31 | 209 | 181 |
| 18 | 26 | 2093 | 122 |
| 19 | 29 | 205 | 66 |
| 20 | 24 | 205 | 97 |
| 21 | 36 | 1046 | 146 |
| 22 | 29 | 297 | 90 |
| 23 | 30 | 260 | 59 |
| 24 | 25 | 233 | 57 |
| 25 | 36 | 619 | 150 |
| 26 | 27 | 1161 | 52 |
| 27 | 34 | 310 | 96 |
| 28 | 29 | 261 | 65 |
| 29 | 42 | 238 | 134 |
| 30 | 36 | 257 | 186 |
| 31 | 28 | 219 | 74 |
| 32 | 30 | 311 | 60 |
| 33 | 42 | 224 | 113 |
| 34 | 29 | 297 | 88 |
| 35 | 29 | 256 | 89 |
| 36 | 31 | 274 | 100 |
| 37 | 24 | 696 | 104 |
| 38 | 31 | 364 | 103 |
| 39 | 26 | 243 | 56 |
| 40 | 38 | 573 | 47 |
| **Number** | **Age (y)** | **Initial Serum CK(U/I)** | **Second Serum CK(U/I)** |
| 41 | 31 | 1761 | 155 |
| 42 | 30 | 539 | 99 |
| 43 | 26 | 838 | 80 |
| 44 | 24 | 238 | 57 |
| 45 | 30 | 211 | 70 |
| 46 | 28 | 259 | 144 |
| 47 | 39 | 200 | 150 |
| 48 | 39 | 211 | 144 |
| 49 | 33 | 474 | 61 |
| 50 | 24 | 203 | 127 |
| 51 | 29 | 290 | 64 |
| 52 | 29 | 313 | 63 |
| 53 | 30 | 294 | 89 |
| 54 | 39 | 219 | 66 |
| 55 | 26 | 229 | 60 |
| 56 | 30 | 300 | 102 |
| 57 | 29 | 319 | 80 |
| 58 | 24 | 330 | 65 |
| 59 | 28 | 227 | 81 |
| 60 | 36 | 591 | 128 |
| 61 | 32 | 257 | 91 |
| 62 | 29 | 280 | 37 |
| 63 | 29 | 288 | 80 |
| 64 | 22 | 276 | 70 |
| 65 | 31 | 354 | 70 |
| 66 | 26 | 230 | 67 |
| 67 | 31 | 314 | 101 |
| 68 | 26 | 297 | 70 |
| 69 | 28 | 240 | 129 |
| 70 | 32 | 268 | 89 |
| 71 | 32 | 318 | 42 |
| 72 | 34 | 271 | 60 |
| 73 | 26 | 306 | 61 |
| 74 | 28 | 207 | 111 |
| 75 | 26 | 226 | 82 |
| 76 | 30 | 219 | 98 |
| 77 | 29 | 401 | 62 |
| 78 | 31 | 883 | 61 |
| 79 | 35 | 256 | 75 |
| 80 | 28 | 487 | 90 |
| 81 | 31 | 388 | 179 |
| 82 | 35 | 208 | 177 |
| **Number** | **Age (y)** | **Initial Serum CK(U/I)** | **Second Serum CK(U/I)** |
| 83 | 34 | 216 | 109 |
| 84 | 25 | 671 | 60 |
| 85 | 28 | 227 | 218 |
| 86 | 33 | 391 | 79 |
| 87 | 29 | 249 | 60 |
| 88 | 23 | 675 | 65 |
| 89 | 34 | 236 | 158 |
| 90 | 27 | 4426 | 82 |
| 91 | 27 | 670 | 106 |
| 92 | 34 | 268 | 91 |
| 93 | 35 | 230 | 74 |
| 94 | 26 | 961 | 118 |
| 95 | 30 | 485 | 59 |
| 96 | 45 | 220 | 174 |
| 97 | 27 | 468 | 55 |
| 98 | 31 | 300 | 64 |
| 99 | 29 | 673 | 61 |
| 100 | 25 | 735 | 69 |
| 101 | 29 | 1507 | 170 |
| 102 | 30 | 1040 | 94 |
| 103 | 26 | 1204 | 70 |
| 104 | 26 | 208 | 76 |
| 105 | 23 | 200 | 194 |
| 106 | 32 | 1128 | 103 |
| 107 | 30 | 661 | 78 |
| 108 | 21 | 302 | 128 |
| 109 | 25 | 250 | 51 |
| 110 | 32 | 219 | 72 |
| 111 | 26 | 363 | 139 |
| 112 | 28 | 239 | 74 |
| 113 | 26 | 6052 | 69 |
| 114 | 32 | 254 | 75 |
| 115 | 27 | 1642 | 77 |
| 116 | 30 | 208 | 84 |
| 117 | 26 | 1149 | 96 |
| 118 | 24 | 249 | 94 |
| 119 | 21 | 275 | 72 |
| 120 | 28 | 342 | 139 |
| 121 | 31 | 3007 | 120 |
| 122 | 39 | 225 | 67 |
| 123 | 37 | 218 | 181 |
| 124 | 23 | 3521 | 75 |
| **Number** | **Age (y)** | **Initial Serum CK(U/I)** | **Second Serum CK(U/I)** |
| 125 | 32 | 205 | 95 |
| 126 | 28 | 382 | 63 |
| 127 | 43 | 276 | 89 |
| 128 | 43 | 203 | 173 |
| 129 | 43 | 206 | 71 |
| 130 | 42 | 306 | 166 |
| 131 | 28 | 785 | 178 |
| 132 | 31 | 203 | 58 |
| 133 | 24 | 800 | 184 |
| 134 | 31 | 203 | 154 |
| 135 | 28 | 86 | 67 |
| 136 | 25 | 385 | 85 |
| 137 | 35 | 220 | 166 |
| 138 | 33 | 229 | 169 |
| 139 | 25 | 2371 | 71 |
| 140 | 31 | 213 | 172 |
| 141 | 26 | 214 | 81 |
| 142 | 32 | 418 | 94 |
| 143 | 47 | 1038 | 142 |
| 144 | 30 | 246 | 124 |
| 145 | 43 | 248 | 83 |
| 146 | 28 | 2106 | 52 |
| 147 | 31 | 202 | 82 |
| 148 | 29 | 479 | 109 |
| 149 | 28 | 230 | 61 |
| 150 | 30 | 246 | 52 |
| 151 | 28 | 245 | 137 |
| 152 | 25 | 312 | 84 |
| 153 | 28 | 220 | 86 |
| 154 | 26 | 211 | 71 |
| 155 | 28 | 1076 | 55 |
| 156 | 26 | 655 | 115 |
| 157 | 38 | 301 | 82 |
| 158 | 29 | 3524 | 95 |
| 159 | 24 | 597 | 86 |
| 160 | 26 | 561 | 81 |
| 161 | 29 | 298 | 152 |
| 162 | 29 | 236 | 100 |
| 163 | 34 | 215 | 108 |
| 164 | 30 | 258 | 95 |
| 165 | 24 | 362 | 109 |
| 166 | 26 | 242 | 118 |
| **Number** | **Age (y)** | **Initial Serum CK(U/I)** | **Second Serum CK(U/I)** |
| 167 | 26 | 272 | 4090 |
| 168 | 26 | 237 | 121 |
| 169 | 32 | 280 | 147 |
| 170 | 30 | 269 | 79 |
| 171 | 26 | 93 | 94 |
| 172 | 29 | 3118 | 81 |
| 173 | 27 | 333 | 41 |
| 174 | 24 | 285 | 89 |
| 175 | 29 | 203 | 60 |
| 176 | 32 | 498 | 68 |
| 177 | 36 | 307 | 83 |
| 178 | 30 | 261 | 177 |
| 179 | 34 | 223 | 58 |
| 180 | 32 | 231 | 98 |
| 181 | 26 | 216 | 198 |
| 182 | 24 | 240 | 154 |
| 183 | 27 | 235 | 104 |
| 184 | 26 | 481 | 99 |
| 185 | 32 | 318 | 94 |
| 186 | 30 | 211 | 134 |
| 187 | 25 | 844 | 73 |
| 188 | 28 | 217 | 68 |
| 189 | 24 | 218 | 109 |
| 190 | 34 | 233 | 65 |
| 191 | 31 | 329 | 79 |
| 192 | 26 | 9121 | 80 |
| 193 | 37 | 233 | 89 |
| 194 | 31 | 238 | 116 |
| 195 | 32 | 273 | 95 |
| 196 | 30 | 228 | 62 |
| 197 | 35 | 2624 | 97 |
| 198 | 25 | 224 | 58 |
| 199 | 28 | 253 | 136 |
| 200 | 24 | 205 | 64 |
| 201 | 32 | 4227 | 127 |
| 202 | 25 | 574 | 138 |
| 203 | 33 | 393 | 71 |
| 204 | 28 | 304 | 122 |
| 205 | 38 | 215 | 149 |
| 206 | 27 | 2278 | 115 |
| 207 | 41 | 1692 | 51 |
| 208 | 26 | 3924 | 123 |
| **Number** | **Age (y)** | **Initial Serum CK(U/I)** | **Second Serum CK(U/I)** |
| 209 | 31 | 1072 | 47 |
| 210 | 32 | 1214 | 54 |
| 211 | 41 | 224 | 89 |
| 212 | 27 | 251 | 87 |
| 213 | 30 | 402 | 166 |
| 214 | 29 | 249 | 144 |
| 215 | 23 | 491 | 126 |
| 216 | 25 | 402 | 165 |
| 217 | 27 | 742 | 90 |
| 218 | 30 | 644 | 93 |
| 219 | 35 | 543 | 123 |
